# Partial Root-Zone Drying of Olive (*Olea europaea* var. 'Chetoui') Induces Reduced Yield under Field Conditions

**DOI:** 10.1371/journal.pone.0157089

**Published:** 2016-06-17

**Authors:** Soumaya Dbara, Matthew Haworth, Giovani Emiliani, Mehdi Ben Mimoun, Aurelio Gómez-Cadenas, Mauro Centritto

**Affiliations:** 1 Centre Régional des Recherches en Horticulture et Agriculture Biologique, Chott Mariem, 4042, BP57, Tunisia; 2 Trees and Timber Institute, National Research Council (CNR—IVALSA), Via Madonna del Piano 10, I-50019, Sesto Fiorentino (FI), Italy; 3 Institut National Agronomique de Tunisie, 43 Avenue Charles Nicolle, Tunis, 1082, Tunisia; 4 Dept Ciencias Agrarias y del Medio Natural, Universitat Jaume I, campus Riu Sec, E-12071, Castellon, Spain; Mediterranean Agronomic Institute at Chania, GREECE

## Abstract

The productivity of olive trees in arid and semi-arid environments is closely linked to irrigation. It is necessary to improve the efficiency of irrigation techniques to optimise the amount of olive fruit produced in relation to the volume of water used. Partial root-zone drying (PRD) is a water saving irrigation technique that theoretically allows the production of a root-to-shoot signal that modifies the physiology of the above-ground parts of the plant; specifically reducing stomatal conductance (*g*_s_) and improving water use efficiency (WUE). Partial root-zone drying has been successfully applied under field conditions to woody and non-woody crops; yet the few previous trials with olive trees have produced contrasting results. Thirty year-old olive trees (*Olea europaea* ‘var. Chetoui’) in a Tunisian grove were exposed to four treatments from May to October for three-years: ‘control’ plants received 100% of the potential evapotranspirative demand (ETc) applied to the whole root-zone; ‘PRD_100_’ were supplied with an identical volume of water to the control plants alternated between halves of the root-zone every ten-days; ‘PRD_50_’ were given 50% of ETc to half of the root-system, and; ‘rain-fed’ plants received no supplementary irrigation. Allowing part of the root-zone to dry resulted in reduced vegetative growth and lower yield: PRD_100_ decreased yield by ~47% during productive years. During the less productive years of the alternate bearing cycle, irrigation had no effect on yield; this suggests that withholding of water during ‘off-years’ may enhance the effectiveness of irrigation over a two-year cycle. The amount and quality of oil within the olive fruit was unaffected by the irrigation treatment. Photosynthesis declined in the PRD_50_ and rain-fed trees due to greater diffusive limitations and reduced biochemical uptake of CO_2_. Stomatal conductance and the foliar concentration of abscisic acid (ABA) were not altered by PRD_100_ irrigation, which may indicate the absence of a hormonal root-to-shoot signal. Rain-fed and PRD_50_ treatments induced increased stem water potential and increased foliar concentrations of ABA, proline and soluble sugars. The stomata of the olive trees were relatively insensitive to super-ambient increases in [CO_2_] and higher [ABA]. These characteristics of ‘hydro-passive’ stomatal behaviour indicate that the ‘Chetoui’ variety of olive tree used in this study lacks the physiological responses required for the successful exploitation of PRD techniques to increase yield and water productivity. Alternative irrigation techniques such as partial deficit irrigation may be more suitable for ‘Chetoui’ olive production.

## Introduction

The production of olives, and products derived from olives, is a major agro-industry in Mediterranean areas with the global market worth over €11 billion per annum [1]. The sustainability of this industry faces a number of converging pressures associated with climate change, population growth and unsuitable agricultural practices [2, 3]. The productivity of olive trees (*Olea europaea* L.) is largely constrained by the availability of water during the summer months when the fruit develops [4]. The majority of European olive groves are currently rain-fed without supplementary irrigation [5]. Global climate models predict that Mediterranean summers will likely become hotter, with an increased frequency and duration of drought events that will coincide with episodes of raised temperatures relative to the norm [6]. Olive trees possess a number of physiological adaptations to cope with drought [7–9]. Nevertheless, longer and more severe droughts may have significant implications for the production of olives [10, 11]. Supplementary irrigation increasing soil water content to field capacity dramatically increases the yield of olives per tree, but also promotes vegetative growth reducing the efficiency of irrigation when measured relative to crop production [12]. The effectiveness of irrigation is gauged by ‘water productivity’: the amount of yield produced per unit of water applied in irrigation [13]. Furthermore, in the future the availability of irrigation water will likely be constrained by increased population levels, industrialisation and urbanisation, combined with the possible effects of climate change on the temporal and spatial distribution of water [14]. It is therefore necessary to optimise the impact of irrigation on yield through development of irrigation technologies based on physiological studies of plant responses to water deficit [15, 16].

The partial root-zone drying (PRD) technique involves applying irrigation to one half of the root-zone whilst the remaining half is allowed to dry [15]. The PRD approach is based on laboratory split-root studies; whereby a plant experiences the physiological effects of water deficit due to the presence of root-to-shoot signals indicating soil drying, but as water uptake is sustained by the irrigated portion of the root-system the physical effects associated with drought, such decreased water potential/content, do not occur [17]. As soil dries, the transport of abscisic acid (ABA) in the xylem increases [18], and this may also be associated with an alteration of pH [19]. These signals induce a number of physiological adaptations within the leaf such as stomatal closure [20], reduced mesophyll conductance (*g*_m_) [21], lower respiration [22] and enhanced expression of antioxidants [22, 23] to conserve water and protect the photosynthetic physiology. The irrigated and drying portions of the root-zone are alternated every 2–4 weeks during PRD, as roots within a drying soil are only able to sustain an ABA ‘drought’ signal for 10 to 15 days [16]. Under field conditions PRD has been successfully utilised in grape (*Vitis vinifera* L.) vineyards, where plants subject to PRD exhibited reduced vegetative growth, no decline in yield and enhanced fruit quality in comparison to plants that received full irrigation to the entire root-system [15, 16]. Potato (*Solanum tuberosum*) when grown under PRD also exhibited lower vegetative growth, but identical tuber yield to plants grown under control conditions that received twice the amount of water [24]. Partial root-zone drying also maintained yield in field grown orange trees (*Citrus sinensis*) irrigated to 60% of the volume of water used in control conditions (PRD_60_) [25, 26], pomegranate (*Punica granatum*) at PRD_75_ [27], apple (*Malus domestica*) at PRD_50-60_ [28] and PRD_50_ [29], mandarin (*Citrus reticulata*) at PRD_50-100_ [30], mango (*Manifera indica*) at PRD_50_ [31], cotton (*Gossypium hirsutum*) at PRD_50-100_ [32], okra (*Abelmoschus esculentus*) at PRD_50_ [33] and maize (*Zea mays*) at PRD_40-80_ [32]. Field trials have also reported reductions in crop yield under PRD associated with lower total water availability (eg. [32, 34]). However, despite reduced yields, crops grown under PRD generally exhibited higher production relative to the total volume of water used in irrigation; possibly making PRD an acceptable technique in areas affected by limited water availability [35]. This may indicate that the PRD technique may improve the efficiency of irrigation by achieving a similar yield with less water.

The development of PRD techniques applicable to a high value crop that occurs in drought prone areas such as olives would confer significant economic and social benefits (eg. [30]). The yield and quality of olive fruit is closely related to water availability during the summer growing season, when precipitation is generally low and potential evapotranspiration is high [4, 36]. To reduce water-loss, the stomata of olive trees close as soils dry and evaporative demand increases [8, 37–39]. In response to water deficit, rates of stomatal (*g*_s_) and mesophyll (*g*_m_) conductance to CO_2_ often decline in unison, these diffusive limitations to the uptake of CO_2_ reduce the concentration of CO_2_ at the site of carboxylation within the chloroplast envelope (*C*_c_) causing a reduction in the rate of photosynthesis (*A*) [40, 41]. However, olive trees in a split-root experiment where one half of the root-system was exposed to a drying soil, while the remainder received the same volume of water as the control plants, exhibited enhanced *g*_m_ values. Increased *g*_m_ levels were not associated with any change in the carboxylation capacity of ribulose-1,5-bisphosphate carboxylase/oxygenase (RubisCO) (*V*c_max_), or the maximum rate of electron transport required for ribulose-1,5-bisphosphate (RuBP) regeneration (*J*_max_) [22]. This may suggest that a root-to-shoot signal [18] induces increased transport of CO_2_ across the mesophyll layer in drought stressed olives [22], thus enhancing the ratio of *g*_m_ to *g*_s_ [42]. Furthermore, olive trees grown in split-root pot experiments exhibited lower leaf water potentials and *g*_s_ when half of the root system was exposed to a drying soil [22, 23, 43, 44], but crucially did not have lower rates of *A* [22].

Pot based split-root studies confer high levels of temporal and spatial regulation of the distribution of water, allowing an in-depth analysis of the physiological responses of olive trees to drying of soil around a section of the root-zone (eg. [22]). However, it is not possible to achieve such a degree of control under field conditions, and as a result the observations of laboratory studies may not be fully replicated in the open field (eg. [9, 44]). Field grown olive trees (var. ‘Manzanilla de Sevilla’) where the root-zone was either totally irrigated or partially allowed to dry, exhibited broadly consistent values of *g*_s_ and *A* between the two treatments [45]. A two-year study into the effects of allowing half of the root-zone to dry (using PRD_100_ and PRD_50_ levels of irrigation) on 11 year-old olive trees (var. ‘Picholine marocaine’) in Morocco observed that under field conditions PRD_100_ plants that received identical water levels to control increased yield, whilst trees receiving half of the amount of water supplied to the control plants (PRD_50_) showed 15–20% decline in yield [46]. This marginally lower yield did not affect oil acidity or polyphenol content of the fruits, which determine the quality of olive oil [47]. The olive plants exposed to PRD_50_ displayed the lowest leaf water potential values, while those of the PRD_100_ plants did not statistically differ from the control. However, levels of *A* and *g*_s_ in the PRD_50_ and PRD_100_ plants were both ~33% lower than the control treatment. The photosynthetic capacity for CO_2_ assimilation, expressed by *V*c_max_ and *J*_max_, was not significantly reduced by PRD_50_, suggesting that using 50% of the required volume of water to replace 100% of the potential evapotranspiration in a PRD system reduced *A* through stomatal closure and not via biochemical limitations [9]. In contrast, drying of a portion of the root-zone of 30 year-old olive trees (var. ‘Manzanilla de Sevilla’) indicated that a PRD of 30% (raised to 100% during pit hardening and prior to harvest) of control water levels was relatively ineffective, inducing minor 5.2 and 11.4% declines in *g*_s_ and *A*, respectively, during June to August [48]. From 2007 to 2009 this PRD resulted in a 41.2% reduction in yield, but had no effect on the dimensions or quality of individual olive fruit [44]. However, field trials of PRD using low-quality saline water irrigation in Tunisian olive groves (var. ‘Chemlali’) to 30% of control irrigation levels induced a slight 11% reduction in olive yield with no effect on fruit oil content [49].

These field studies exposing part of the root-system to a drying soil indicate that PRD irrigation with reduced volumes of water do induce some reduction in the yield of olive trees (eg. [44, 46, 49]). However, the extent of any decline in yield and the underlying physiological causes are unclear. In drought affected plants, *A* and yield are often related to diffusive limitations to the transport of CO_2_ [40, 41]; yet in the field grown olive trees subject to PRD there were contrasting observations of the strength of any relationship between *g*_s_ and yield (eg. [44, 48, 49]). The variation in these observations may be due to differences in the amount of water used in the PRD irrigation systems. For example PRD_100_ treatment provides 100% of ETc to one half of the root-system, thereby meeting all of the water requirements of the olive trees while simultaneously providing a root-to-shoot signal that may modify physiological and morphological growth responses of the olive trees. Whereas supplying a lower volume of water to the irrigated portion of the root-zone may induce a more pronounced drought response associated with lower overall water availability (eg. [9, 49]).

In this study we conducted a field based investigation into the effects of two different PRD irrigation levels (PRD_100_ and PRD_50_) in comparison to control (full ETc irrigation to both sides of the root-zone) and rain-fed (no supplementary irrigation) growth conditions on 30 year-old olive trees (var. ‘Chetoui’) in Tunisia. The aims of this study were to: i) investigate the effect of PRD on both stomatal and mesophyll conductance to CO_2_ and biochemical limitations to CO_2_ uptake, and their relationship to *A*; ii) characterise any potential relationships between *g*_s_, *g*_m_ and *A* with the quality and quantity of olive fruit and oil produced by trees under different levels of PRD; iii) gauge the impact of differential PRD on the growth of olive trees, specifically whether enhanced vegetative growth may limit the effectiveness of supplementary irrigation in terms of fruit yield, and; iv) identify whether PRD is an effective irrigation technique in terms of the yield achieved on the basis of the amount of water supplied during irrigation, and the physiological and morphological mechanisms that underpin this response.

## Materials and Methods

### Experimental site and irrigation treatments

The study was conducted in the experimental farm of the National Agronomic Institute of Tunisia, located in the Mornag plain, 15 Km south east of Tunis (Latitude 32°7, Longitude 10°14). The olive trees were 30 year-old trees belonging to the ‘Chetoui’ variety, which is the most important cultivar for olive oil production in the North of Tunisia. The olive grove had not previously been irrigated prior to the instigation of the study. The occurrence of alternate bearing of fruit in olive trees strongly affects production on a year-to-year basis [50]. The present study was conducted over a three-year period (2005 to 2007) consisting of two more-productive ‘on-years’ and one less-productive ‘off-year’. Measurements of gas-exchange and biochemical analysis of leaves and olive fruit took place in the final on-year of the study in 2007. At the beginning of summer in May, four irrigation treatments were applied on the basis of potential evapotranspiration (ETc) calculated using the formula:

$$\text{ETc}=K_{c}*\text{ETo}$$ (1)

where ETo is the reference evapotranspiration calculated from the Penman-Monteith equation [51] and *K*_c_ is the crop factor (monthly values of 0.6 during June–September and 0.65 during October-November) [52]. Weather data was recorded each day at a weather station within the experimental farm and used to estimate ETo [51]. Values of monthly rainfall are given in Table 1. The olive trees were subjected to four irrigation treatments: control trees received full irrigation with 100% of Etc to both sides of the root system; PRD_100_ irrigation supplied 100% of the volume of water required to meet ETc to one half of the root system, with the irrigated and drying halves of the root-zone alternated every ten days; the PRD_50_ irrigation treatment provided 50% of the volume of water equivalent to ETc to one side of the root-system, alternated between sides every ten days, and; rain-fed plants received no supplementary irrigation. To provide the olive trees with water, a drip irrigation system was utilised. Emitters were placed at a distance of 0.5 m from the trunk. The discharge rate for each emitter was 8 dm^3^ h^-1^, with a total of eight emitters used for each tree in the control and PRD_100_ treatments (distributed according to whether water was applied to the whole or part of the root-zone), and four emitters per tree in the PRD_50_ treatment. Water was provided to the olive trees from May until October. Trees were arranged in a randomised block design of twelve trees per block with three replicate blocks for each of the four irrigation treatments.

**Table 1:** Monthly rainfall in mm during the study. Irrigation was performed during May to October each year.

	Jan	Feb	Mar	April	May	June	July	Aug	Sept	Oct	Nov	Dec	Total
2005	55	103	38	39	8	10	3	28	42	30	19	105	480
2006	148	48	31	19	35	0	0	0	27	82	50	178	618
2007	10	55	120	20	32	12	0	0	4	122	56	73	504

### Stem water potential measurement

Midday stem water potential (Ψ_s_) was measured using a Scholander pressure chamber (PMS Instrument Company, Albany, Oregon, USA) during October 2007. Stem water potential was determined on leaves enclosed in a black plastic bag covered with aluminium foil for two hours prior to measurement. Three stems of 15cm in length were analysed to produce a mean Ψ_s_ value for each plant, with the average of three replicates then taken to represent mean Ψ_s_ for each irrigation treatment. Measurements were performed between 12:00 and 13:00 hours.

### Gas exchange and fluorescence measurements

Leaf gas exchange and fluorescence parameters of the central leaf section were simultaneously measured using a LI-6400-40 leaf chamber fluorometer (Li-Cor, Inc., Nebraska, USA) equipped with a 2 cm^2^ cuvette during October 2007 (the most important period for olive fruit development prior to harvesting at the end of November). Measurements were performed on the youngest fully expanded leaf of at least two branches from each tree, with the mean of three trees taken to represent the value for a given treatment. The measurements were made between 10:00 and 15:00 hours at a saturating photon flux density (PPFD) of 1400 μmol m^-2^s^-1^, [CO_2_] of 380 ppm, leaf temperature of 25°C and relative humidity ranged between 45 and 55%. Instantaneous transpiration efficiency was expressed as the ratio of *A* to *g*_s_. Mesophyll conductance was calculated using the variable J method involving simultaneous measurements of gas-exchange and chlorophyll fluorescence parameters as described by Harley et al. [53]. The CO_2_ compensation point to photorespiration (Γ*) was measured by increasing *C*_i_ at four different levels of photosynthetically active radiation (400, 300, 200 and 100 μmol m^-2^ s^-1^)[54]. Levels of respiration in the light (*R*_d_) were analysed using the Kok method [55]; and respiration in the dark (*R*_n_) was measured by switching off the light in the cuvette, when CO_2_ release from the leaf had become stable for approximately five to 10 minutes. This was recorded and considered to represent *R*_n_ [41]. Values of Γ* and *R*_d_ used in the calculation of *g*_m_ utilising the variable *J* method are given in Table 2. Total conductance to CO_2_ (*g*_tot_) was calculated as:

$$g_{\text{tot}}=\left[g_{s}*g_{m}\right]/\left[g_{s}+g_{m}\right]$$ (2)

**Table 2:** Photon flux density saturated (1400 μmol m^-2^s^-1^) photosynthesis (*A*), stomatal conductance (*g*_s_), mesophyll conductance (*g*_m_), total conductance (*g*_t_), instantaneous transpiration rate (ITE), light respiration (*R*_L_), dark respiration (*R*_D_), maximal fluorescence yield (*F*_v_/*F*_m_) and stomatal density (SD) of control, PRD_100_, PRD_50_ and rain-fed *Olea europaea* ‘Chetoui’ trees. Values are means of eight to twelve plants per treatment. ± indicates one standard error. Means followed by different letters indicate significant difference (*P* < 0.05) using a one-way ANOVA with LSD *post-hoc* test.

	*A* (μmol m^-2^s^-1^)	*g*_s_ (mmol m^-2^s^-1^)	*g*_m_ (mmol m^-2^s^-1^)	*g*_t_ (mmol m^-2^s^-1^)	ITE (mmol mol^-1^)	*R*_d_ (μmol m^-2^s^-1^)	*R*_n_ (μmol m^-2^s^-1^)	*F*_v_/*F*_m_	SD (stomata / mm^2^)
Control	11.44 ± 0.44^c^	0.106 ± 0.005^b^	0.198 ± 0.010^b^	0.069 ± 0.003^b^	4.70 ± 0.29^a^	0.76 ± 0.07^b^	1.35 ± 0.11^b^	0.778 ± 0.006^a^	608 ± 122^a^
PRD_100_	12.22 ± 0.60^c^	0.099 ± 0.005^b^	0.194 ± 0.003^b^	0.066 ± 0.002^b^	6.95 ± 0.19^c^	0.77 ± 0.03^b^	1.42 ± 0.18^b^	0.780 ± 0.013^a^	576 ± 115^a^
PRD_50_	9.45 ± 0.47^b^	0.087 ± 0.003^a^	0.157 ± 0.017^a^	0.051 ± 0.003^a^	5.53 ± 0.40^b^	0.57 ± 0.05^a^	1.10 ± 0.06^a^	0.767 ± 0.006^a^	620 ± 124^a^
Rainfed	8.15 ± 0.54^a^	0.080 ± 0.005^a^	0.120 ± 0.022^a^	0.045 ± 0.004^a^	4.45 ± 0.33^a^	0.46 ± 0.11^a^	0.95 ± 0.09^a^	0.768 ± 0.010^a^	570 ± 114^a^

Photosynthetic response curves to increased [CO_2_] were conducted in the field using the method of Centritto et al. [56]. These *A*/*C*_i_ curves were performed at a standard leaf temperature of 25°C and a higher temperature of 30°C. The Farquhar et al. [57] model of C3 photosynthesis was used to calculate values of *V*c_max_ and *J*_max_ following Ethier and Livingston [58].

### Leaf biochemical analysis

Leaves were collected from the olive trees at the same time as the leaf gas exchange measurements were conducted and immediately frozen in liquid nitrogen and then storeed at -80°C prior to analysis. Total soluble sugars were quantified following the phenol-sulfuric acid method [59] using a spectrophotometer (Jenway 6505UV/VIS, Bibby Scientific, Staffordshire, UK) at 490 nm and D-glucose as standard. Proline was determined spectrophotometrically following the ninhydrin method of Bates et al. [60] at a wavelength of 520nm from the organic phase using toluene as a blank. The abscisic acid (ABA) content of leaves was measured using high-performance liquid chromatography (Alliance 2695, Waters Corporation, Milford, Massachusetts, USA). Hormone quantification was monitored with a mass spectrometer (Quattro LC, Micromass Ltd, UK) [61].

### Olive yield, oil quality and olive tree growth parameters

Olive fruits were harvested by hand at the same phenological stage when the fruits had matured at the end of November. The Maturity Index was 5 according to Mailer et al. [62], indicating that the majority of the fruit had a colouring that was black with more than 50% purple flesh [63]. The yield of olive fruit of the nine trees monitored for each treatment was weighed using a field balance. Yield was then expressed as kg per hectare. The olives were crushed using a laboratory scale mill to extract their oil. To assess the quality of the olive oil: acidity was determined following Wolf [64]; polyphenols were measured spectrophotometrically at 727 nm using Folin-Denis reagent [65], and chlorophyll content of the oils was measured using a spectrophotometer at 630, 670 and 710 nm [66].

After the olive fruit harvest, vegetative growth was evaluated by measuring the shoot length and leaf surface area. Twenty vegetative and fruit bearing shoots evenly distributed around the circumference of each tree were selected. Ten leaves were then chosen for area analysis from each shoot. Leaf surface area was measured using a digital planimeter (CID 203 LEASER). Measurements of stomatal density values of the mid-section of each leaf were performed by preparing nail varnish ‘negatives’ of the abaxial leaf surface. These were then placed onto glass microscope slides and the number of stomata per unit leaf area determined using an Olympus (B07, BH-2, Olympus, Tokyo, Japan) microscope equipped with an Olympus camera (B06, C-35AD-2, Olympus, Tokyo, Japan). Stomatal density was measured from 27 leaves per treatment (nine leaves for each tree), with the number of stomata being counted for three images for each leaf [67].

### Statistical analyses

Statistical analyses were performed using SPSS 10 (IBM, New York, USA). To test the effect of irrigation treatment on physiological, biochemical and morphological parameters a one-way ANOVA with LSD *post-hoc* test was used to assess differences in variance between samples.

## Results

### Leaf gas-exchange

**Fig 1:**
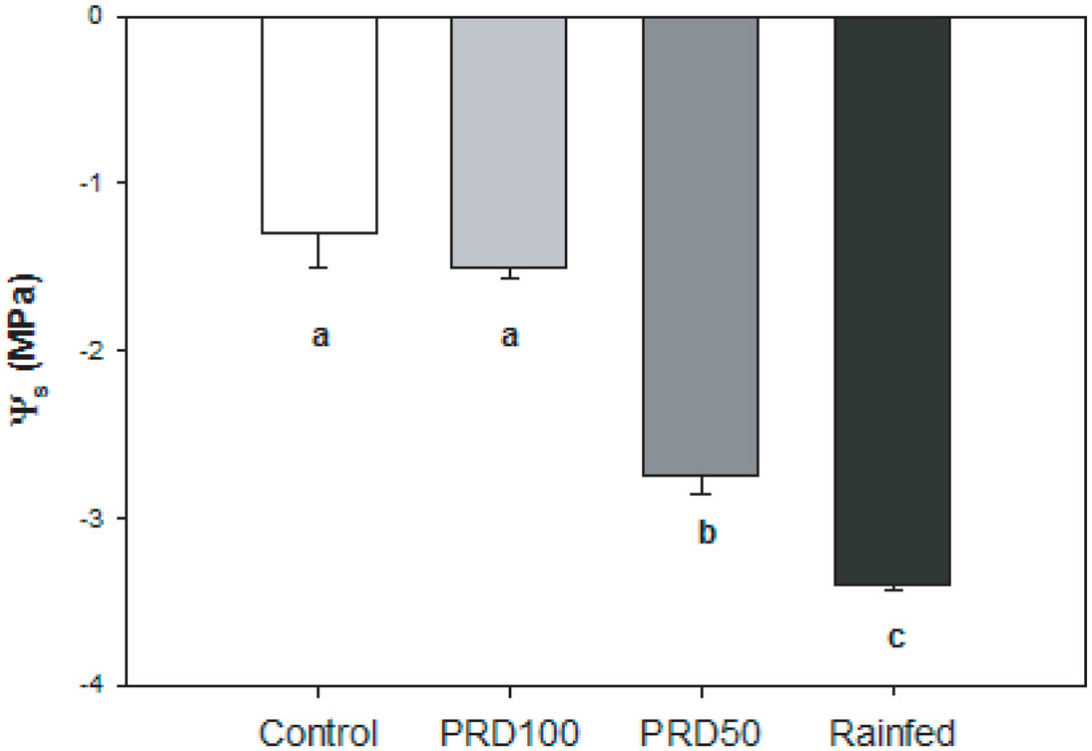
Midday stem water potential (Ψ_s_) of olive trees (var. ‘Chetoui’) grown under control, PRD_100_, PRD_50_ and rain-fed irrigation treatments in October 2007. Error bars indicate one standard error either side of the mean. Letters indicate significant difference (P < 0.05) using a one-way ANOVA with LSD *post-hoc* test.

**Fig 2:**
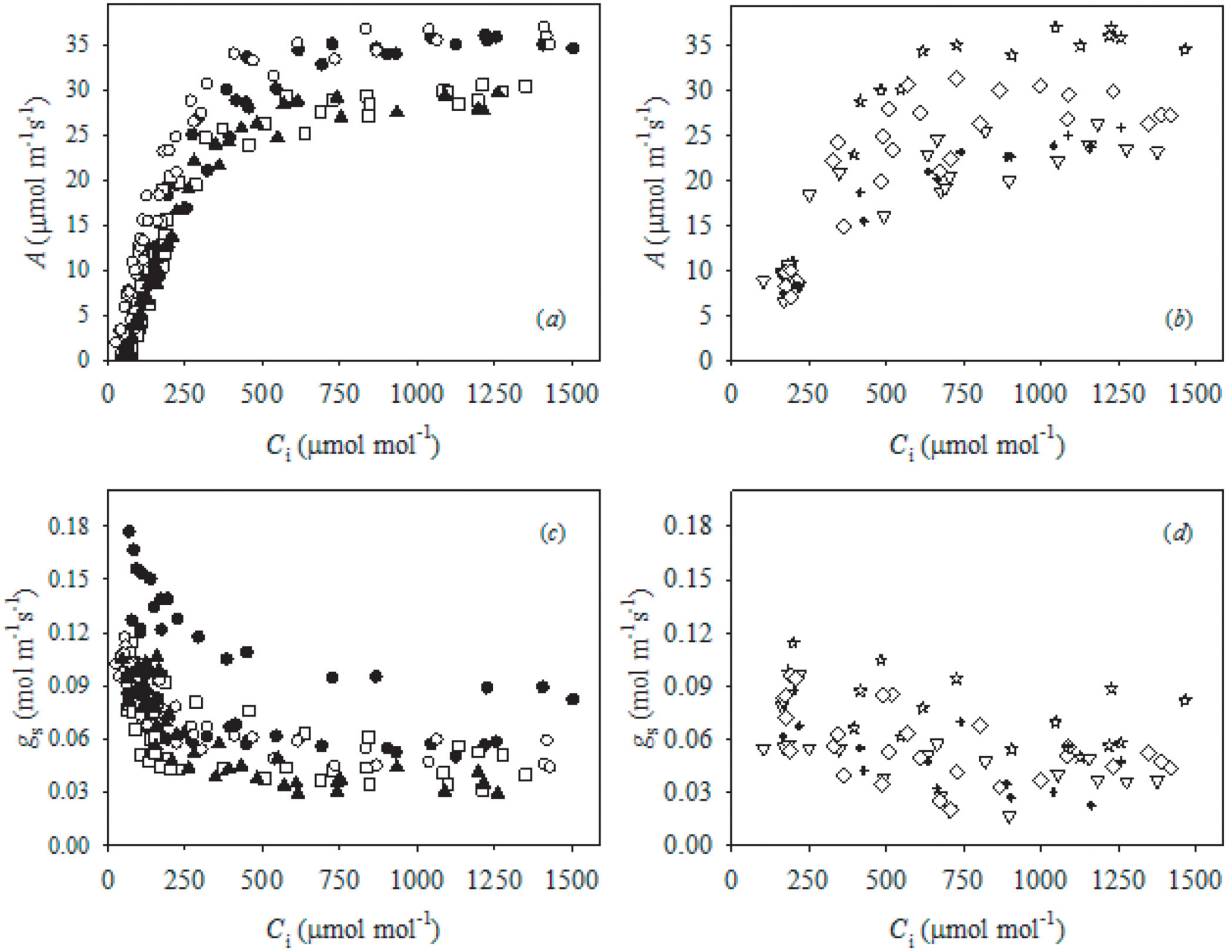
The relationship between (*a*, *b*) photosynthesis (*A*) and intercellular [CO_2_] (*C*_i_), and (*c*, *d*) stomatal conductance (*g*_s_) and *C*_i_ measured after exposing olive (var. ‘Chetoui’) leaves to a [CO_2_] of ~50 ppm for approximately 60 minutes to force stomatal opening [56] during the morning (between 900 and 1100 h), with relative humidity ranging between 45 and 55% and leaf temperature of 25°C(*a*, *c*), and during the early afternoon (between 1330 and 1430 h) with relative humidity ranging between 30 and 35% and leaf temperature of 30°C (*b*, *d*). The measurements were made on three to four plants per irrigation treatment in saturating PPFD (1400 μmol m^-2^s^-1^) in the control (●,☆), PRD_50_(□,▿), PRD_100_ (○,◇) and rain-fed (▲,✚) treatments during October 2007.

**Fig 3:**
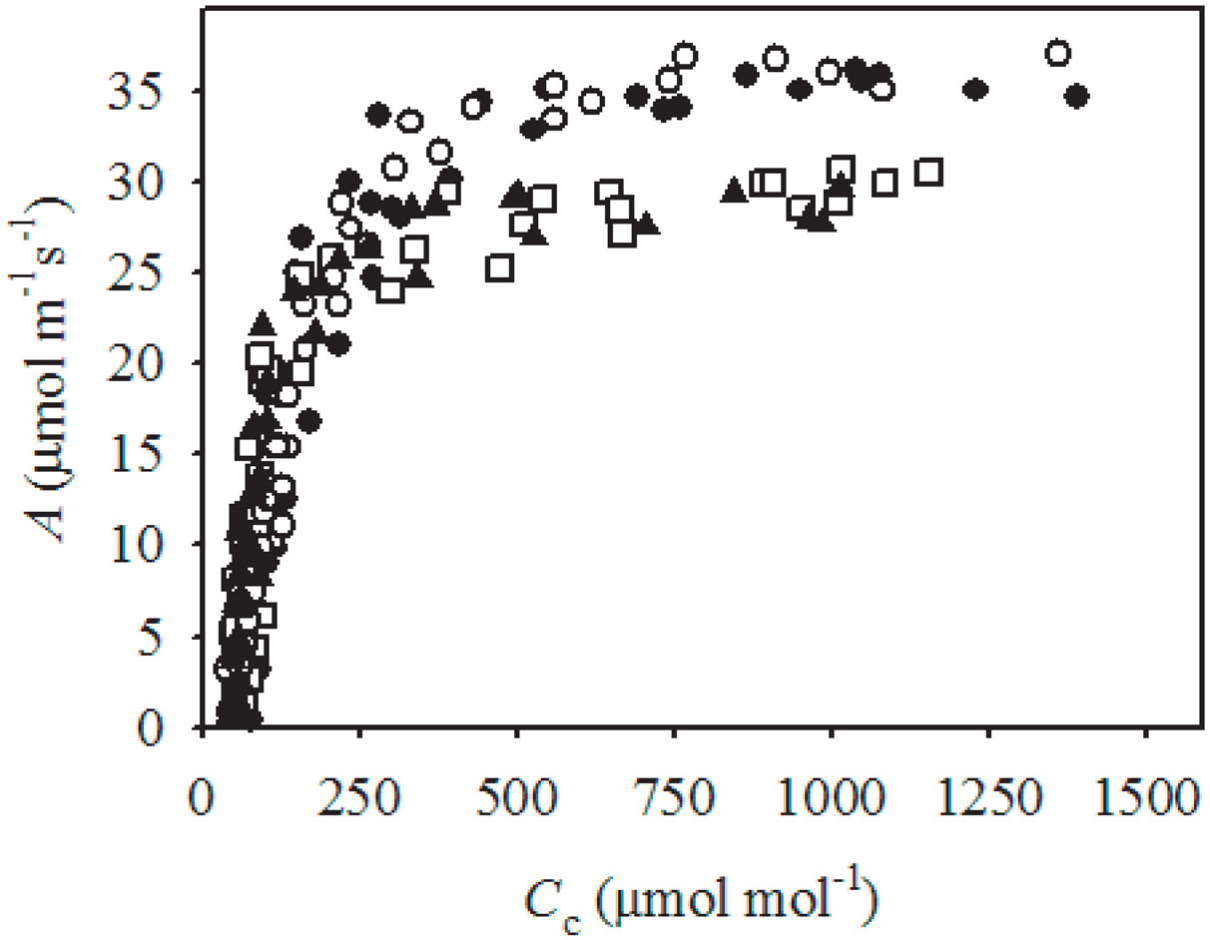
The relationship between photosynthesis (*A*) and chloroplastic [CO_2_] (*C*_c_) measured after exposing olive (var. ‘Chetoui’) leaves to a [CO_2_] of ~50 ppm for approximately 60 minutes to force stomatal opening (Centritto *et al*., 2003) during the morning (between 9:00 and 11:00 h). The measurements were made on three to four plants per water treatment, in saturating PPFD (> 1200 μmol m-^2^s^-1^), with relative humidity ranging between 45 and 55%, and leaf temperature of 25°C in the control (●), PRD_50_ (□), PRD_100_ (○) and rain-fed (▲) treatments.

Partial root-zone drying reduced *g*_s_ values in both PRD_100_ (-6.7%) and PRD_50_ (-17.9%) treated trees compared to control olive trees (Table 2). Stomatal conductance in the PRD_100_ plants was not statistically different to levels observed in the control plants, and the Ψ_s_ values of control and PRD_100_ plants were also identical. Olive trees grown under PRD_50_ (-115.4%) and rain-fed (-169.2%) conditions exhibited significantly reduced Ψ_s_, that corresponded to the lowest values of *g*_s_ recorded in the study (Table 2 and Fig 1). Mesophyll and total conductance to CO_2_ followed similar patterns to *g*_s_; being highest under control conditions and lowest under the PRD_50_ and rain-fed treatments. Rates of photosynthesis were slightly, but not significantly, higher in the PRD_100_ than control treatment; however, *A* declined alongside *g*_tot_ in the PRD_50_ and rain-fed treatments (Table 2). The marginally higher values of *A* obtained under PRD_100_ than the control treatment were not associated with biochemical capacity to assimilate CO_2_ (Fig 2a and Table 3). Irrigation treatment did not affect *V*c_max_ but declines in *J*_max_ were observed in PRD_50_ and rain-fed trees. Furthermore, the small differences in *V*c_max_ and *J*_max_ between olive trees receiving full ETc (control and PRD_100_) and those receiving lower amounts (PRD_50_ and rain-fed) became less apparent when the effect of *g*_m_ on movement of CO_2_ was taken into consideration, and the relationship between *A* and *C*_c_ plotted (Fig 3). The increase in *A* with *C*_i_ and *C*_c_ is less pronounced in PRD_50_ and rain-fed plants, suggesting that biochemical in addition to diffusive limitations to *A* occur under these conditions (Figs 2a and 3). An increase in cuvette temperature enhanced the apparent treatment effects on the biochemical efficiency of CO_2_ assimilation. At the higher temperature, rain-fed and PRD_50_ grown plants exhibited declines in *V*c_max_ and *J*_max_. Furthermore, *V*c_max_ and *J*_max_ values of PRD_100_ plants were also reduced in comparison to control levels, suggesting that PRD irrigation reduced the capacity for CO_2_-uptake at higher leaf temperatures (Fig 2b). The *g*_s_ values of olive trees under all irrigation treatments showed a decline as *C*_i_ was increased at sub-ambient concentrations. Increases of *C*_i_ to levels above ambient did not induce further reductions in *g*_s_ (Fig 2c). The efficiency of photosystem II (*F*_v_/*F*_m_) was broadly consistent in trees grown under all treatments, indicative of the adaptation of olive to environments characterised by high evaporative demand, high levels of PAR and low water availability (Table 2). Partial drying of the root-zone induced significant increases in leaf level instantaneous transpiration rate (ITE) relative to the control and rain-fed treatments (Table 2).

**Table 3:** Analysis of *A/C*_i_ curves in Fig 2a based on the Farquhar et al. (1980) model of C3 photosynthesis following Ethier and Livingston (2004) to calculate the carboxylation capacity of ribulose-1,5-bisphosphate carboxylase/oxygenase (RubisCO) (*Vc*_max_), the maximum rate of electron transport required for ribulose-1,5-bisphosphate (RuBP) regeneration (*J*_max_) and their ratio. Values are the mean of three response curves. ± indicates one standard error either side of the mean. Means followed by different letters indicate significant difference (*P* < 0.05) using a one-way ANOVA with LSD *post-hoc* test.

	*V*c_max_ (μmol m^-2^ s^-1^)	*J*_max_ (μmol m^-2^ s^-1^)	*V*c_max_: *J*_max_
Control	92.4 ± 4.6^a^	170.8 ± 1.4^a^	0.541 ± 0.024^a^
PRD_100_	91.0 ± 15.6^a^	159.9 ± 5.9^a^	0.566 ± 0.082^a^
PRD_50_	75.5 ± 2.5^a^	142.2 ± 3.2^b^	0.531 ± 0.006^a^
Rain-fed	66.8 ± 1.3^a^	136.2 ± 5.0^b^	0.491 ± 0.011^a^

### Water potential and biochemical effects of partial root-zone drying

**Fig 4:**
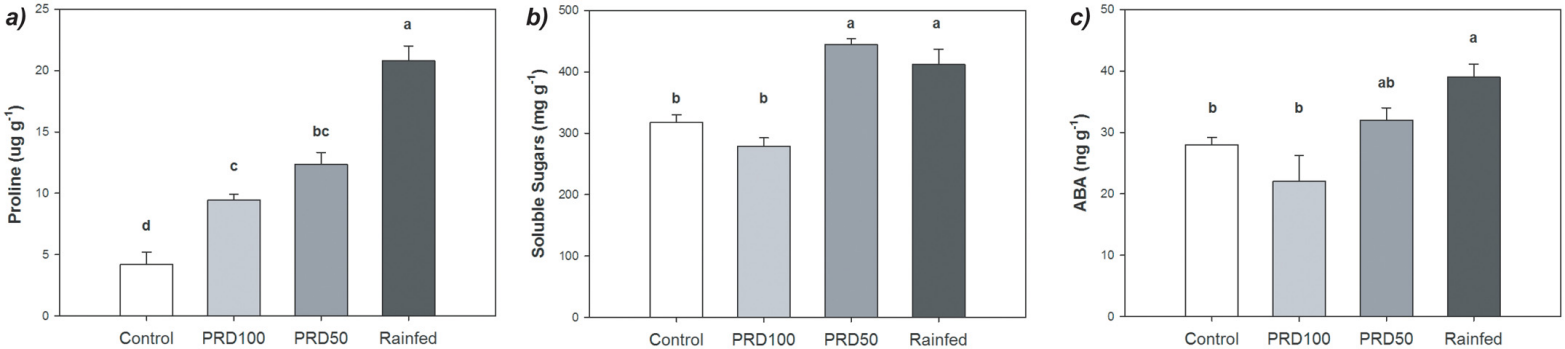
Regulators of leaf osmotic status in olive trees (var. ‘Chetoui’) exposed to control, PRD_100_, PRD_50_ and rain-fed irrigation treatments in October 2007: Foliar concentration of a) proline; b) soluble sugars, and c) abscisic acid (ABA). Error bars indicate one standard error either side of the mean. Letters indicate significant difference (P < 0.05) using a one-way ANOVA with LSD *post-hoc* test.

The lower Ψ_s_ observed in olive plants grown under PRD_50_ and rain-fed conditions may be the result of osmotic adjustment in the trees exposed to lower levels of water availability (Fig 4). The lower yield of the PRD100. The concentration of leaf soluble sugars was also elevated in the PRD_50_ and rain-fed treatments relative to the control and PRD_100_ (Fig 4b); replicating the patterns observed in Ψ_s_ induced by the irrigation treatments (Fig 1). Levels of foliar [ABA] were 21.3% lower in the PRD_100_ treatment than in the control. Leaf ABA concentration in PRD_50_ plants was marginally 14.3% higher than their control counterparts; a significant increase in [ABA] (39.3%) was only observed in plants under the rain-fed treatment (Fig 4c).

### Effect of partial root-zone drying on growth and yield

**Fig 5:**
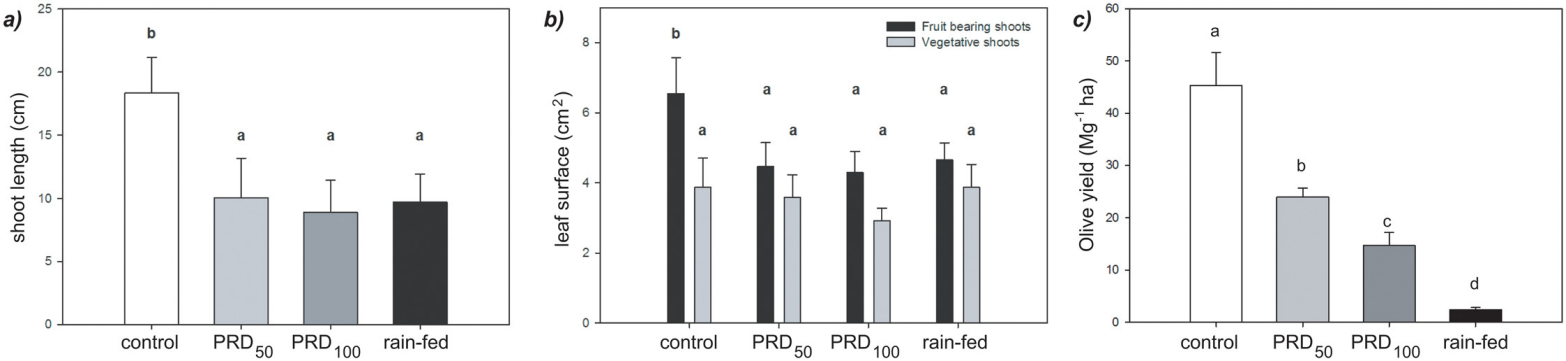
Growth effects of control, PRD_100_, PRD_50_ and rain-fed irrigation treatments on olive trees (var. ‘Chetoui’): a) shoot length in 2007; b) leaf surface area of vegetative and reproductive shoots in 2007, and; c) mean olive yield in the two productive ‘on-years’ 2005 and 2007. Error bars indicate one standard error either side of the mean. Letters indicate significant difference (P < 0.05) using a one-way ANOVA with LSD *post-hoc* test.

Allowing part of the root-zone to dry significantly altered the growth patterns of the 30 year-old olive trees. Shoot length was significantly reduced in the rain-fed and both of the PRD treatments (Fig 5a). Likewise, the leaf area of the fruit bearing shoots was also reduced in the PRD and rain-fed treated plants. However, alteration in the level and spatial distribution of irrigation did not alter leaf area on vegetative shoots; suggesting that PRD irrigation may affect reproductive tissues predominantly over vegetative growth (Fig 5b). Critically, this is borne out in the yield of the trees during the ‘on-years’ of 2005 and 2007; control irrigation to both sides of the root-zone to a level sufficient to replace potential evapotranspiraton resulted in the highest yield of olive fruit of 45.3 Mg ha^-1^; however, PRD_100_ induced a significant 47.0% reduction in yield to 24.0 Mg ha^-1^. Partial root-zone drying utilising 50% of the level of water applied to the control plants induced an -67.6% decline in yield to 14.7 Mg ha^-1^; while the lowest yield of 2.4 Mg ha^-1^ occurred in the rain-fed treatment, 5.3% of the yield achieved under full control irrigation (Fig 5c). During the ‘off-year’ of 2006, yield was reduced in all treatments and supplementary irrigation did not influence the production of olive fruit (Table 4). The amount of oil produced per kg of olive fruit was unaffected by the irrigation treatment, as was the quality of the oil with the acidity occurring below the 0.8% level required to be classified as ‘extra-virgin’. The total polyphenol content of the oils was relatively high [68], also consistent with extra-virgin standards of the International Olive Council [69] and suggestive of a high degree of oxidative stability [70]. The amount of chlorophyll in the oil is indicative of the maturity of the olives; the higher the concentration of chlorophyll, the less ripe the olives at the time of harvest [71]. The oil produced by plants subject to all treatments showed no significant effects of irrigation on chlorophyll concentration (Table 5). Nonetheless, values observed in this experiment were generally higher than values reported by other studies [44, 47, 71].

**Table 4:** Olive fruit yield of olive trees (var. ‘Chetoui) grown under control, PRD50, PRD100 and rain-fed irrigation treatments in the three years of this study. Olive trees generally alternate between productive ‘on years’ and less productive ‘off years’; 2005 and 2007 were more productive ‘on’ years. Values indicate the mean of nine trees per treatment. ± indicates standard error. Letters after the value indicate significant difference using a one-way ANOVA with LSD *post-hoc* test.

Treatment	Yield (Mg^-1^ ha)		
	2005	2006	2007
rain-fed	2.700 ± 0.475^a^	1.114 ± 0.102^a^	2.067 ± 0.733^a^
PRD_50_	9.443 ± 1.107^ab^	1.943 ± 0.187^a^	19.900 ± 1.790^bc^
PRD_100_	25.650 ± 2.676^c^	1.759 ± 0.540^a^	22.333± 2.126^bc^
control	47.167 ± 13.268^d^	1.018± 0.073^a^	43.422 ± 4.638^d^

**Table 5:** The percentage yield of olive oil per unit of olive fruit, and the quality of olive oil extracted from fruit grown under control, PRD100, PRD50 and rain-fed treatments. Olive oil quality was gauged by acidity (Kalua et al., 2007), polyphenol content (Tsimidou et al., 1992; Aparicio et al., 2013) and the amount of chlorophyll remaining within the oil from the skin of the oil fruit (Salvador et al., 2001). Values are means of eight to twelve plants per treatment. ± indicates one standard error. Means followed by different letters indicate significant difference (*P* < 0.05) using a one-way ANOVA with LSD *post-hoc* test.

Treatments	Oil concentration (%)	Acidity (%)	Polyphenol (ppm)	Chlorophyll (ppm)
Control	16 ± 0.20^a^	0.10±0.05^a^	472.43 ± 37.64^b^	1.86 ± 0.60^a^
PRD100	16 ± 0.20^a^	0.10±0.05^a^	416.56 ± 54.38^b^	1.33 ± 0.67^a^
PRD50	15 ± 1.41^a^	0.30±0.07^a^	462.84 ± 13.70^b^	1.80 ± 0.29^a^
Rain-fed	16 ± 0.20^a^	0.35±0.07^a^	540.03 ± 24.99^a^	1.12 ± 0.51^a^

## Discussion

The majority of olive groves are rain-fed, particularly those in hilly areas where water for irrigation is either expensive or impractical. Irrigation with relatively low volumes of water (70–200 mm^3^ ha^-1^ per week) can increase yields to 80% of those of plants supplied with sufficient water to replace ETc [4, 72]. In 2010, within the EU ~40% of Spanish, 26% of Italian and ~36% of Greek olive groves were irrigated; with irrigated trees responsible for 52% of olive fruit production [5]. However, the availability of fresh-water for irrigation will likely be constrained by population growth, urbanisation and industrialisation, combined with the potential effects of climate change on precipitation patterns [73]. This necessitates the optimisation of water-use in irrigation techniques, often termed ‘*more crop per drop*’ [13]. Partial root-zone drying has been successfully applied to numerous crops (see summary in introduction) and to olives grown in split-pot experiments (eg. [43]). However, the results of this and previous studies (eg. [44, 48]) suggest that PRD may not be as effective in certain varieties of olive trees under field conditions, or it may not be possible to achieve sufficiently rigorous control of the distribution of water under field conditions.

### Photosynthesis and diffusive conductance to CO_2_

Leaf area photosynthetic rates were unaffected by the PRD_100_ treatment relative to control irrigation; however, halving of the volume of water supplied to the plant in the PRD_50_ treatment reduced *A* by 17.4%, with levels of *A* 28% lower in rain-fed than control plants (Table 2). In contrast to previous studies, this reduction in *A* induced by PRD was not solely the result of diffusive limitations (eg. [9, 22]), but a combination of reduced *g*_tot_ and decreased biochemical uptake of CO_2_, as indicated by lower values of *V*c_max_ and *J*_max_ (Table 3). These declines in *V*c_max_ and *J*_max_ become more pronounced at higher temperatures, exacerbating the effect of drought on the carbon-uptake of olive trees through increased photorespiration (Fig 2b) [74]. The higher yield of the control plants may be related to their greater photosynthetic area (Fig 5), as allometric relationships have been observed between leaf biomass and yield [75], possibly due to correlations between whole plant photosynthetic rates, total leaf area and yield [76]. Higher vegetation growth is often associated with increased levels of respiration to fulfil the energetic requirements of metabolic processes [77]. Olive trees grown under rain-fed and PRD_50_ conditions exhibited respective ~31 and ~26% lower levels of respiration in the light and dark than their counterparts receiving the full volume of water required to meet ETc; potentially accounting for their lower vegetative growth and fruit production (Fig 5) (eg. [78]).

As the availability of water in soil declines, stomatal closure occurs to reduce transpiration and limit water-loss from the plant [38]. In a split-root experiment involving bean (*Phaseolus vulgaris*) stomatal closure occurred as a result of a root-to-shoot ABA signal indicating soil drying prior to any reduction in leaf water potential [79]. A reduction in *g*_s_ did not occur in the olive tree subject to PRD_100_ irrigation (Table 2), possibly indicating that a hormonal root-to-shoot signal did not occur or was not inducing stomatal closure (eg. [80–82]). A degree of stomatal closure occurred in the PRD_50_ and rain-fed treatments; but this was a relatively minor reduction in *g*_s_ of 20–25% (Table 2). The lower values of *g*_s_ in the PRD_50_ and rain-fed olive trees corresponded to lower Ψ_s_; possibly indicating that leaf water content and not a hormonal root-to-shoot signal of soil drying affected *g*_s_ values of olive trees under field conditions (eg. [48]). The osmotic adjustment responsible for the lower Ψ_s_ in PRD_50_ and rain-fed olive trees may be the result of increased concentration of proline [83] and soluble sugars [84]. In addition to the regulation of osmotic potential, proline may play a protective role in the response of olive trees to drought and temperature stress [85].

Stomatal and mesophyll conductance concomitantly decline following drought stress (eg. [41]). The purpose of stomatal closure is to reduce the loss of water from the leaf to the external environment; however, the functional significance of a reduction in the rate of transport of CO_2_ across the mesophyll is less clear [86]. An increase in the ratio of *g*_m_ to *g*_s_ would theoretically improve plant photosynthetic performance under drought conditions [42]. Indeed, under PRD_100_ conditions in a split-root pot experiment, olive trees exhibited a 63% increase in *g*_m_ relative to *g*_s_; potentially indicative of a root-to-shoot signal altering the biochemical properties of the mesophyll layer to the transport of CO_2_ [22]. However, in this study under field conditions *g*_m_ and *g*_s_ were unaffected under PRD_100_, and the *g*_m_ to *g*_s_ ratio remained constant under both PRD treatments. The ratio of *g*_m_ to *g*_s_ did decline by ~20% in rain-fed olive plants, suggesting that the overall lower level of water availability in the rain-fed treatment reduced CO_2_ transport to the chloroplast envelope (Table 2).

The relatively constrained reductions in *g*_s_ values observed in the PRD_50_ and rain-fed plants (Table 2) may be somewhat surprising given the well-documented adaptations of olive trees to drought stress [7]; in particular evidence of stomatal responsiveness to drought [8, 36]. However, PRD_50_ resulted in a mean 5.2% reduction in *g*_s_ values of 35 year-old olive trees [48], suggesting that physiological stomatal closure may not be the result of a root-to-shoot signal of soil drying. Stomatal conductance of water vapour showed similar patterns to increased *C*_i_ in olive trees under all irrigation treatments (Fig 2c). Stomatal conductance declined markedly to sub-ambient increases in *C*_i_, but remained constant as *C*_i_ was increased above ambient levels; contrasting to the hypothesised evolutionary response of angiosperm stomata to above ambient increases in [CO_2_] (cf. [87]), and further evidence to support the lack of a phylogenetic pattern in stomatal responses to CO_2_ [67, 88]. Furthermore, increased foliar [ABA] in the PRD_50_ and rain-fed olive plants did not alter stomatal sensitivity to [CO_2_], despite being considered a defining characteristic of angiosperm stomatal physiology (cf. [89]) and being observed in rose (*Rosa hybrid*) [90]. Not all angiosperms may possess the physiological responses required for PRD to be successful. A split-root study of bell pepper (*Capsicum annuum* L.) found that *g*_s_ was not regulated by a root-to-shoot chemical signal, but stomatal closure occurred in a ‘hydro-passive’ fashion (ie. where guard cell turgor and stomatal opening follow the water status of the whole leaf) related to soil water potential in both root compartments [80]. The absence of evidence indicative of a hormonal root-to-shoot signal of soil drying, or stomatal response to super-ambient [CO_2_] and increased foliar [ABA] may suggest that the varieties of olive used in this study (var. ‘Chetoui’) and others (eg. [44, 48]) lack the physiological capacity to rapidly alter *g*_s_ in response to environmental signals through ‘hydro-active’ stomatal control (ie. where guard cell turgor and stomatal opening are rapidly modified by an influx/efflux of ions/metabolites) (eg. [88, 91]). In essence, the physiological mechanisms required for the successful implementation of a PRD irrigation strategy may not be present in these olive varieties.

Analysis of the most widely grown Tunisian olive cultivars found that Chemlali exhibited greater stomatal control and was more tolerant of drought than Chetoui [37]. The results of the present study indicate that PRD was an ineffective irrigation method in the Chetoui variety; whereas, the yield of the more drought resistant Chemlali variety was only reduced 11% during PRD_30_ irrigation [49]. This may suggest that physiological differences between olive varieties may account for differential responses to PRD irrigation treatments. Stomatal physiological behaviour may vary in olive varieties (eg. [37, 92]) between those that are dominated by ‘hydro-passive’ and ‘hydro-active’ stomatal physiology [93]. This may also offer a mechanistic basis to account for the contrasting results achieved in PRD studies involving olive trees and other angiosperm crops (see summary in introduction), and the comparative success of partial deficit irrigation techniques when applied to olive groves [72].

### Effect of PRD on olive yield and fruit quality

The aim of PRD techniques is to increase the yield of olive fruit per unit of water used in irrigation. Partial root-zone drying has been successfully applied to other crops under field conditions; however, the results of this trial indicate that the PRD approach may be less effective in the Chetoui variety of olive trees. Critically, the yield of olive fruit grown under PRD_100_ during the two ‘on-years’ analysed in this study was ~47% lower than the trees subject to the same level of irrigation under control conditions; while PRD_50_ resulted in a 67.6% reduction in yield, suggesting that the volume of water received by olive trees and the spatial distribution of water determine yield [48]. The reduction in yield induced by PRD_100_ found in this study appears to be at the upper end of decreases in yield observed in previous investigations utilising identical levels of irrigation between the control and PRD treatments that recorded declines of ~51% [44], ~20% [46] and ~11% [49]. The lower yield of the PRD_100_ grown olive trees may be the result of lower xylem flux acting as a hydraulic signal of soil drying (eg. [80]). The increase in foliar concentrations of soluble sugars that occurs during drought has been associated with reduced yield, as the export of photosynthate from the leaf is reduced, thus reducing Ψ_s_ [94]. However, Ψ_s_ (Fig 1) and the concentration of soluble sugars (Fig 4b) were identical under control and PRD_100_ irrigation treatments, suggesting that impaired transport of sugars from the leaf were not responsible for the reduced yield observed under PRD_100_ in this study. Supplementary irrigation during the lower productivity ‘off-year’ did not affect yield; raising the possibility that the effectiveness of water application may be improved over a two-year cycle by withholding irrigation during the non-productive phases of the alternate bearing cycle. This would also decrease vegetative growth during the ‘off-year’, reducing the requirements for pruning or water to sustain the additional leaf area during the following productive ‘on-year’. Nevertheless, reducing irrigation levels during the ‘off-year’ may potentially adversely affect production in the ‘on-year’ if the plants experienced stress that subsequently impaired growth; an aspect that should be determined in future studies of irrigation efficiency.

Despite the lack of evidence of a root-to-shoot signal affecting *g*_s_ in the PRD_100_ olive trees, the reduction in shoot length and leaf area indicate that exposing a portion of the roots to drying soil did effect plant growth (Fig 5a and 5b), and possibly supressing investment in reproductive tissues [95]. However, increased root-to-shoot ABA signals in grapevine promoted reproductive growth, resulting in enhanced yield [96]. Nonetheless, different selective pressures may have resulted in a dissimilar response in olive, where as a comparatively long-lived woody tree, allocation of photosynthate to reproductive growth is reduced under water deficit [97].

The quality and quantity of the oil produced from olive fruit was unaffected by the irrigation treatment (Tables 4 and 5). The olive oil was of a comparatively high standard with low acidity and high levels of polyphenols required for classification as ‘extra-virgin’ [68, 69, 98]. The lack of effect on the characteristics of the oil [44, 49] and the amount of oil produced for a given amount of fruit [44, 47, 49] under PRD irrigation in comparison to control irrigation found in this study is consistent with previous reports. Olive fruit grown under rain-fed conditions has been observed to contain a higher proportion of oil than their irrigated counterparts [44, 49]; a similar response was not observed in the present study, where the percentage oil content of olive fruit was identical under all treatments (Table 4).

The results of this study indicate that PRD irrigation was relatively ineffective in enhancing the yield of olive fruit relative to the volume of water utilised in irrigation (Fig 5c). This may be due to a comparative lack of control afforded under field conditions in isolating part of the root-zone to allow the soil to dry; nonetheless, field trials of PRD have been successful in other woody trees (eg. [25, 27–30]). The results of this study suggests that the Chetoui variety of olive used may lack the necessary physiological responses [37] fundamental to a successful PRD irrigation strategy; whereby a root-to-shoot signal of soil drying affects photosynthetic, leaf gas exchange and osmotic behaviour to improve WUE [16, 17]. The absence of pronounced stomatal closure (Table 2) or active physiological stomatal behaviour to [CO_2_] (Fig 2c) or [ABA] (Fig 4c) may indicate that stomatal control in this variety of olive is largely hydro-passive [88], and the signalling network required for a split-root system to induce stomatal closure and increased water use efficiency is not present (eg. [80]). These findings may suggest that PRD is not suitable for Chetoui variety olive groves; irrigation of the entire root-zone may be more effective in maximising yield through the optimisation of water productivity (eg. [44, 48]). Regulated deficit irrigation to the whole root-zone may be a more effective approach when applied to olive groves, as small volumes of supplementary irrigation have been shown to produce significantly enhanced yield [72].

## Conclusions

Partial root-zone drying has been utilised to improve the water productivity of numerous crops. The successful application of PRD to olives would permit the optimisation of yield relative to water-use in a crop grown in drought prone areas. However, while the results of laboratory based split-root studies of olive trees have been promising; the efficacy of PRD irrigation in the field has been equivocal. In this study, during productive ‘on-years’, yield was significantly reduced by 47% in the PRD_100_ treatment relative to the control, despite receiving the same volume of water. Yield was 68 and 95% lower in the PRD_50_ and rain-fed treatments. The yield of fruit relative to the amount of water used was significantly lower under PRD in comparison to application of water to the whole root-zone. Supplementary irrigation did not enhance olive fruit yield during the less productive ‘off-year’, suggesting that co-ordination of the supply of water with the alternate bearing cycle may enhance water-productivity on a two-year basis. The quality and quantity of oil produced by equal amounts of olive fruit from each irrigation treatment was identical. Lower *A* was observed in the PRD_50_ and rain-fed treatments due to higher diffusive (Table 2) and biochemical (Fig 3) constraints to CO_2_-uptake. A similar pattern was not observed in the PRD_100_ treatment, possibly indicating that a root-to-shoot signal inducing stomatal closure was not present. Stomatal conductance was identical in the control and PRD_100_ treatments, as were Ψ_s_ and foliar [ABA]. Stomatal closure occurred in the PRD_50_ and rain-fed olive trees, with a relatively small reduction in *g*_s_ of 19–29%, which corresponded to lower Ψ_s_ and higher concentrations of the osmotic regulators ABA, proline and soluble sugars (Fig 4). The lack of clear active physiological stomatal behaviour to [CO_2_] (Fig 2c) and [ABA] (Fig 4c) may indicate that the dominant component of stomatal control in the Chetoui variety of olive trees is hydro-passive. The physiological mechanisms required to produce a root-to-shoot signal of soil drying and then induce stomatal closure to enhance the WUE of photosynthesis, may be absent in the Chetoui variety of olive tree; thus constraining the effectiveness of PRD in optimising the water productivity of irrigation. Nonetheless, the required physiological mechanisms for successful application of the PRD technique may be present in other olive varieties. The apparent absence of physiological mechanisms required for PRD in Chetoui olive may negate the effectiveness of PRD in Chetoui olive groves. Periodic deficit irrigation of the entire root-zone may be a more successful approach in optimising crop yield and water productivity in olive trees than applying water to part of the root-system.
